# Location Mechanism and Perception Scheme of Remote Transmission Equipment Layout in Smart Water

**DOI:** 10.1155/2022/6013816

**Published:** 2022-08-26

**Authors:** Shikai Xing, Xiang Xie

**Affiliations:** School of Economics and Management, Beijing Jiaotong University, Beijing, China

## Abstract

Whether the traditional water equipment is equipped with remote transmission unit depends on whether the power consumption of the installed equipment can meet the expected life cycle. In the paper, by using intelligent strategies to save the power consumption of sending data, more sites can be selected to install remote transmitting units. In the process of transmitting data packet sequence, the remote transmission device needs to perceive the environment, interact with the environment and make decisions, and adjust the strategy according to the effect of the decision actions. Therefore, in this paper, the transmission process is modeled as Markov sequence decision model. And the real time signal interference noise ratio of the channel and the transmission delay of the data packet are defined as the state space. The decision action space consists of immediate transmission and delayed one, and the minimum total power consumption is taken as the objective function. The model is solved by Proximal Policy Optimization (PPO) algorithm, and the optimal decision sequence of site selection threshold is obtained.

## 1. Introduction

Different from solving multi-device game problems through reinforcement learning, solving single-device decision-making problems through reinforcement learning can be modeled as a Markov decision process [1]. When the state transition probability of a single device is known, the iterative equation of dynamic programming can be used to solve its decision-making problem. Due to the wireless environment of the installation point of water equipment, its state transition probability is often unknown, and reinforcement learning algorithm can explore the environment through trial and error, and obtain a good strategy to maximize the overall expected return. In this paper, a deep reinforcement learning algorithm will be used to solve the Markov sequence decision problem of a single intelligent device.

## 2. Literature Review

In recent years, wireless communication technology is developing rapidly, especially the Internet of Things communication technology represented by NB-IoT (Narrowband Internet of Things) [2], which has the characteristics of deep coverage, low power consumption, and low remote maintenance cost, which is very suitable for the water industry, the deployment location, data transmission, battery power supply, and other characteristics of the remote transmission equipment. With the full coverage of the NB-IoT network, a large number of IoT devices have been installed or replaced in the urban water supply and drainage network and copy collection system. The number of remote transmission equipment such as water meters and manhole cover monitors installed with NB-IoT modules in urban pipeline networks and copy collection systems is also rapidly increasing.

These remote transmission devices have intelligent features such as on-demand data reporting, intelligent sleep and access to the platform, and online detection with the platform. These intelligent devices are mainly composed of three modules, namely, data acquisition and calculation module, compression and storage module, and data transmission module. These modules are located in the application layer, storage layer, and communication layer of the smart device, respectively.

According to the agreement between the device and the platform [3], the device collects data at a fixed frequency every day, and the amount of data accumulated in each time period is basically fixed. The sensors of the device collect data according to certain rules and frequencies. The basic data is the measurement data of the user's water flow. In addition, there may be additional data such as water pressure and water temperature. The collected data is stored in the remote transmission device. Metering and sensing accumulated data to the platform in the form of incremental or absolute amount through the communication unit on the equipment at a predetermined time every day. The data accumulated during upload will be split into multiple packets and sent.

After the data is sent on the same day, in order to save power consumption and prolong the working life, the remote transmission device will power off itself to sleep or enter the PSM (power save mode) state after sending data, and wake up on the next day or when data is sent, the unsent data will continue to be sent the next day.

Most of the equipment of intelligent water affairs is installed in the weak coverage area of wireless channels such as corridors and wells. On the coverage benchmark, there will also be various frequency shifts, random disturbances generated by interference, and superposition of noises, such as white noise, occlusion, and co-frequency interference. The result of superposition makes the channel quality change randomly.

Therefore, the channel quality of wireless communication is jointly determined by signal coverage and noise interference. Generally, two quantities are used to evaluate the channel quality: RSRP (Reference Signal Receiving Power) and SINR (Signal to Interference plus Noise Ratio).

## 3. Problem definition

Most of the equipment in the urban pipe network system and the copying system are deployed in underground pipelines or corridors. The remote transmission equipment is battery powered and power consumption sensitive. Due to building block reflection or stray interference, the wireless signal will be greatly attenuated, and the battery life is very dependent on the installation environment and data transmission strategy. If the installation location and transmission strategy are not good, some batteries will be consumed quickly from the start of the remote transmission equipment to the use of the battery, which cannot support the data collection and transmission business needs of a period of 6–10 years. Since the installed equipment is unattended, when the equipment needs to replace the battery, it generally needs to be manually operated on-site. When the battery is manually replaced on-site or the traditional manual reading is used, it is often difficult to enter the home and the equipment maintenance cost is relatively high.

Due to the relatively high labor cost of replacing batteries for these devices, from the perspective of investment and cost, it is expected that these devices can maintain a longer life cycle and send more data, thus resulting in the problem of installation and location selection of remote transmission devices.

The installation site selection of the remote transmission equipment depends on two aspects [4]: the wireless signal coverage quality of the installation point, and the ability of the equipment to perceive the installation environment.

Before remote transmission equipment is installed, it is necessary to measure the wireless signal coverage quality at the installation site to ensure compliance with a certain threshold. The determination of the threshold is related to the ability of the device to perceive the installation environment. That is, the stronger the device's ability to perceive the installation environment, the lower the requirements for the quality of wireless signal coverage; on the contrary, the weaker the device's ability to perceive the installation environment, the higher the requirements for the quality of wireless signal coverage. By improving the device's ability to perceive the environment, the requirements of the device on the quality of on-site signal coverage at the installation point are reduced, so that more installation points have the conditions to install remote transmission equipment.

When the channel state changes, the SNR fluctuates. It will affect the power (power consumption) consumed by the remote device when sending data. When the signal gets worse, it takes longer to send data and requires more power consumption. The relationship between power consumption and signal quality presents a nonlinear relationship, that is, when the signal quality gradually deteriorates, the power consumption gradually increases. When the signal quality falls below a certain threshold, the power consumption starts to increase rapidly.

Based on the above-mentioned nonlinear relationship between channel quality and power consumption, there is no clear judgment value, and it is necessary to determine the best action value that should be taken according to the environment and a certain strategy. That is, the remote transmission device detects the channel quality of the wireless communication through the sensor to determine whether to send the data immediately or temporarily. Under the condition of a certain battery capacity of the remote transmission equipment, after all data of the day is sent correctly, the power consumption is the smallest, so that the service life of the remote transmission equipment is the longest. Therefore, when the life cycle of the equipment is 6–10 years, it is necessary to determine the location range of whether the remote transmission unit can be installed according to the amount of data to be sent.

Therefore, the installation site selection and perception improvement of remote transmission equipment is essentially an optimization problem for smart equipment to adopt a certain decision-making mechanism in an unknown environment to minimize the power consumption of the equipment.

## 4. System Model

In this paper, the environment-aware decision-making process of data packets sent by remote equipment is modeled as MDP [5] (Markov decision process) to describe the state transition of the system. And the derivation of the process is equivalent to the layout and location selection mechanism of intelligent water remote transmission equipment.

Remote device is divided into *n* data packets each time, the data packet set *T*_*i*_={*t*_1 _, *t*_2_,…, *t*_*n*_}, is a sample of task distribution, and each task sample corresponds to an MDP process. This MDP process is defined by a quintuple {*S*, *A*, *P*, *R*, *γ*}, where *S* is the state space, *A* is the action space, *P* is the state transition matrix, and *R* is the state transition benefit, which *γ* is the forward benefit discount.

### 4.1. Channel Status

Remote transmission device sends each data packet in turn. According to the agreement, the amount of data sent is fixed. However, due to the random characteristics of wireless channels, the channel quality will vary randomly. Some packets need to be resent if poor channel quality results in higher BER or packet loss.

The signal-to-noise ratio (SINR) of the channel obeys a small-scale Rayleigh distribution, and its probability density function [6] is expressed as

(1) $$\begin{array}{c} p\left(snr\right)=\frac{1}{\bar{snr}}\exp\left(-\frac{snr}{\bar{snr}}\right). \end{array}$$

Among them, *snr*  the threshold value set of SINR is $\bar{snr}$ expressed, and the expectation of SINR is expressed. The NB-IoT network *snr* divides the channel quality into *N* grades according to the value, and sets a total of *N* -1 signal quality thresholds, *snr*={*snr*_1_, *snr*_2_,…，*snr*_*N*−1_}. According to the *snr* threshold, the channel quality can be divided into N states, *C*={*c*_0_, *c*_1_,…*c*_*N*−1_}. The channel state probability is

(2) $$\begin{array}{c} p_{c}\left(c_{i}\right)=\int_{snr_{i}}^{snr_{i+1}}p\left(snr\right)d\left(snr\right). \end{array}$$

Assuming that the next moment of the channel can only be transferred to the adjacent interval, the state transition probability of the channel [6] is

(3) $$\begin{array}{c} p_{c}\left(c_{i},c_{i+1}\right)=\frac{N\left(snr_{i+1}\right)*T_{f}}{p_{c}\left(c_{i}\right)}，\;i\in \left\{0,1,\ldots ,N-2\right\},\\ p_{c}\left(c_{i},c_{i-1}\right)=\frac{N\left(snr_{i}\right)*T_{f}}{p_{c}\left(c_{i}\right)}，\;i\in \left\{1,2,\ldots ,N-1\right\}. \end{array}$$

Among them, *N*(*snr*_*i*_) =  $\sqrt{2*\pi *snr_{i}/\bar{snr}}$, *∗*, $f_{D}*\text{exp}\left(-snr/\bar{snr}\right)$, *f*_*D*_ is the maximum Doppler frequency shift.

### 4.2. Device Latency Status

Device latency status of the device is described by the total delay after the device sends the *i-th* data packet, and the total delay corresponds to the power consumption. Suppose the sending time of the first data packet is, and the sending time of *t*_0_ the *i-th* data packet is *t*_*i*_. Obviously, the total delay after the device transmits the *i-th* data packet is not only *related* to the immediate decision, but also depends on the delay accumulation of the previous *i*−1 data packets. The solution of the total transmission delay of the *i-th packet* can be transformed into a combination of immediate decision-making and solution of the total delay of the *i-1-th* packet.

(4) $$\begin{array}{c} \;\tau_{i}=\tau_{i-1}+\Delta \tau_{i}+T_{f}, \end{array}$$

where the device latency status is *τ*={*τ*_1_,…, *τ*_*n*_},  Δ*τ*_*i*_ denote the waiting time determined by the immediate decision is related to the channel state and *τ*_*i*−1_ depends on the state of the previous *i* -1 data packets, *T*_*f*_ is a constant which is the transmission time of one data packet.

### 4.3. System Status

System state *S* can be defined as the combined state of the channel state and the device delay state, i.e., *S*≜*C* ⊗ *τ*. The above equation is a recursive process and cannot be solved by dynamic programming because the transition probability is unknown.

### 4.4. Action

*A* denotes the remote transmission device taken when the new data packet is to be transmitted and it can be expressed as *A*≜{0,1}: When *a* = 0, it means to transmit immediately; when *a* = 1, it means to suspend transmission.

## 5. MDP Process Analysis of Remote Equipment

### 5.1. System State Transition

When the remote transmission device sends data packets in sequence in a queuing manner, according to the channel state, the remote transmission device may choose to send the data packets immediately. This strategy works in the connected state, corresponding to the instantaneous SINR, and uses the transmit power to transmit with a certain probability. And transfer to the new channel state and device delay state.

The remote device may also adopt a policy of suspending sending a packet. This strategy waits in an idle state, expecting a better channel state to transmit, and the smart device Δ*τ*_*i*_ continues to transmit after an interval according to the change of the channel state. After waiting for transmission, the system state transitions to the new channel state and in-device delay state. Since each data packet is small, the time to transmit a data packet is very short compared to the waiting time, which is defined as one frame *T*_*f*_.

Δ*τ*_*i*_ set as the single-step delay time of the *i* -th data packet, which is randomly determined Δ*τ*_*i* _ by the intelligent device according to the environmental state. It is the pause waiting time. This waiting will cause the overall delay of the time for the device to complete the transmission of all data packets Δ*τ*_*i* _. *τ*_*i*_ is the total delay of sending the first *i* data packets. *t*_*i*_ In order to send the time to be transmitted for the *i* - *th* data packet, the delay before the i-1 data packet *t*_*i*−1_ − *t*_0_ is to be transmitted is

(5) $$\begin{array}{c} \tau_{i-1}=\left\{\begin{array}{cc} t_{i-1}-t_{0}+T_{f}, & \text{sentting the}\left(i-1\right)\text{th Packets immediately,}\\ t_{i-1}-t_{0}+\Delta \tau_{i-1}+T_{f},\; & \text{suspending the }\left(i-1\right)\text{th packet,} \end{array}\right) \end{array}$$

the *ith* data packet is decided, the recursive formula of the delay state of the device is

(6) $$\begin{array}{c} \tau_{i}=\left\{\begin{array}{cc} \tau_{i-1}+T_{f}, & \text{sentting the ith Packets immediately,}\\ \tau_{i-1}+\Delta \tau_{i}+T_{f}, & \text{suspending the ith packet.} \end{array}\right) \end{array}$$

Set *τ*_0_=0. Then, the one-step delay of the *i* -th packet is

(7) $$\begin{array}{c} \Delta \tau_{i}=\left\{\mathrm{int}\left[\frac{N-snr_{i}\;}{\;\left(snr_{N}-snr_{1}/N\right)}\right]\right\}*\delta . \end{array}$$

*SINR* is related to the delay. The better the *SINR*, the smaller the delay. *δ* In order to suspend the parameter of the minimum time segment, this paper assumes that the channel quality remains unchanged in the minimum time segment to ensure that the retransmission is an incoherent channel, and *N* is the number of states. And *int* is a rounding operation function.

### 5.2. System Benefits and Costs

After the device sends a data packet, if it does not receive the confirmation message from the platform within a certain period of time, it will resend the data packet. Due to the different acknowledgment mechanisms of different platforms, the additional power consumed by the device to resend the data packet is different. Therefore, an equivalent model is established: in different environments, the device increases the transmit power to ensure the same bit error rate during the transmission and transmission of data packets.

NB-IoT network adopts two rates to transmit data according to the *SINR*, corresponding to BPSK modulation (Binary Phase Shift Keying) and Q PSK modulation (Quadrature Phase Shift Keying). When the channel quality is relatively poor, the NB - IoT network uses the BPSK modulation method to obtain the minimum transmit power consumption when the bit error rate requirements are met under different channel states [7],

(8) $$\begin{array}{c} P_{ber}\left(s_{i}\right)\leq 0.5*erfc\left(\sqrt{\frac{\left(snr_{i}*P_{i}^{\prime }\right)}{\sigma }}\right), \end{array}$$

where *P*_*ber*_(*s*_*i*_)=*ber*_*i*_/*σ* is the bit error rate probability and $erfc\left(x\right)=2/\sqrt{\pi }*\int_{0}^{x}e^{-\eta^{2}}\text{d}\eta$ is the Gaussian error function.

*Ber* is the allowable bit error rate of the wireless channel, *σ* is interference noise power. It is the *P*_*i*_′ equivalent transmit power that guarantees no packet loss or retransmission when the bit error rate is met. When the channel quality is relatively good, the NB-IoT network uses the QPSK modulation method to obtain the minimum transmission power consumption when the bit error rate requirements are met under different channel states, namely,

(9) $$\begin{array}{c} P_{ber}\left(s_{i}\right)\leq 0.2*exp\left(\frac{-1.6*snr_{i}*P_{i}^{\prime }}{3*\sigma }\right). \end{array}$$

When the device suspends sending the first *i* data packet, the power consumption is *I*_0_*∗*Δ*τ*_*i*_+*P*_*i*_′/*v∗T*_*f*_, where *I*_0_ is the idle state working current of the device, and when the device is in the waiting state, the idle state current of the *I*_0_ device within Δ*τ*_*i* _ the time is. *v* is the device voltage and *T*_*f*_ is the length of time in the connected state to transmit a data packet.

When the device sends immediately, Δ*τ*_*i*_=0, the power consumption of a single packet is

(10) $$\begin{array}{c} \;W_{i}=\Delta \tau_{i}*I_{0}+\frac{P_{i}^{\prime }}{v}*T_{f}. \end{array}$$

After sending each data packet of all samples, the total power consumption of one day can be expressed as

(11) $$\begin{array}{c} \;W_{\text{day}}=\sum_{m}^{M}\sum_{i=1}^{n}\left(\Delta \tau_{i}*I_{0}+\frac{P_{i}^{\prime }}{v}*T_{f}\right). \end{array}$$

Among them, *M* is the number of samples sent in one day, each sample is the data to be sent once agreed in the protocol, and *n* is the number of data packets corresponding to one sample. The goal of the system is to find the most efficient decision to send packets for maximum benefit, i.e., minimum total power consumption.

Defined *t*_*i*_ immediate reward function is the negative value of the power consumption of a single data packet, that is, *R*_*i*_=−*W*_*i*_, the immediate reward function of the amount of data sent per day is the negative value of the total power consumption, i.e., *R*_day_=−*W*_day_.

### 5.3. Location Range of Equipment

The expected life days that the remote device can use is *d*, and the battery capacity is set to be *W*_total_, then *d*=*W*_total_/*W*_day_, the expected life years *D*=*d*/365.

If the remote device only has data to be sent once a day, the location decision can be calculated by the following formula

(12) $$\begin{array}{c} W_{\text{total}}=\sum_{j=0}^{d}\sum_{i=1}^{n}\left(\Delta \tau_{i}*I_{0}+\frac{P_{i}^{\prime }}{v}*\tau_{0}\right). \end{array}$$

Since the battery capacity of the device *W*_total_ is a known value, when the expected number of days or years of use is given, an optimal environment-aware decision chain needs to be obtained to obtain the optimal Δ*τ*_*i*_ sum *P*_*i*_′ scheduling.

Under the condition that the battery capacity, expected service life, and data volume of the device are all determined, whether a remote transmission device can be installed at an installation point depends entirely on Δ*τ*_*i*_ and *P*_*i*_′ there is a feasible solution. In order to delay the service life of the equipment, it is also necessary to seek the optimal strategy *A* to obtain the optimal solution.

According to the data collection and transmission rules of the device and the platform, each time data is sent every day during actual operation, it will be divided into *n* data packets and sent in a sequence. During the sample learning period, data can be sent continuously for *M* times, parameter training can be performed, battery power consumption can be measured, and it can be converted into whether the battery life meets the expected number of years.

## 6. Optimal Decision-Making Based on Deep Reinforcement Learning

For the above MDP problem, this paper will use deep reinforcement learning algorithm to solve. In the random and aligned MDP process, the state *s* and the decision action *a* have randomness and can *π*(*a|s*) be represented by a conditional distribution, representing *π* the mapping from the state to the decision action *a*, that is, the policy. The following defines a class of neural networks to fit policies.

### 6.1. Sequence Representation of Neural Networks

**Figure 1:**
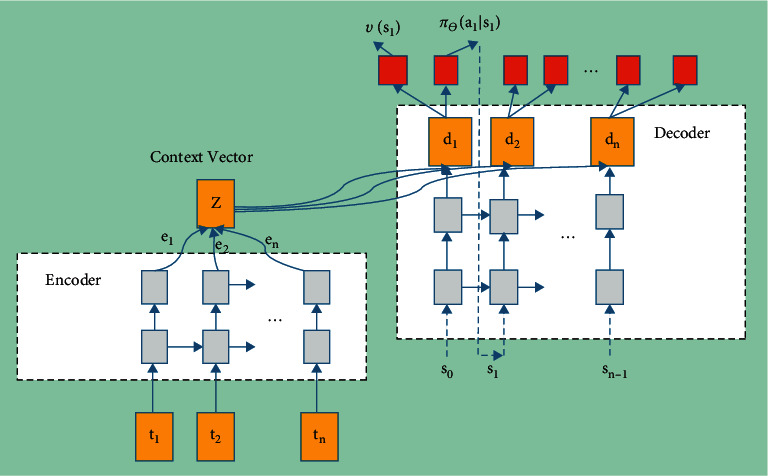
Architecture of the seq2seq neural network.

The smart device divides the data sent each time into *n* data packet sequences to send, and needs to make a sending decision on the *n* data packets in turn to form a decision sequence. This paper uses seq2seq (sequence-to-sequence) [8] neural network to express the above process sequence, that is, input a sequence and output a sequence. The network structure is shown as Figure 1.

Seq2Seq neural network consists of an encoder and a decoder, both of which are R NN networks (Recurrent Neural Network, recurrent neural network). Assuming that the parameters of the neural network are *θ*, then when the state *s* is input, the conditional probability of outputting the optimal decision *a* can be rewritten *π*_*θ*_(*a|s*). According to the input *t*_*i*_, the neural network first encodes the learning and memory through the encoder, and then outputs through the decoder according to the memory of the network *d*_*j*_, and then passes through two different activation functions, corresponding to the output state value function *v*(*s*) and decision sequence probability *π*_*θ*_(*a|s*).

In order to reflect the characteristics of the data to be sent, the sequence of data packets can be converted into a sequence of embedded vectors and input to the neural network. Input *t*_*i*_ = [data packet sequence number *i*, data packet size], which is a 2-dimensional vector. For different types of devices, the amount of data to be sent each time may be different, so the size of the data packet is different. The size of the last data packet of each device is also different from the previous *n*−1 data packets, which is the margin of the data amount divided by *n*−1.

Denote the encoder and decoder [9] as^,^*f*_*dec*_, respectively, *f*_*enc*_, then.

The output of the encoding part is

(13) $$\begin{array}{c} e_{i}=f_{enc}\left(t_{i},e_{i-1}\right). \end{array}$$

The output of the decoding part is

(14) $$\begin{array}{c} d_{j}=f_{dec}\left(z_{j},s_{j-1},a_{j-1}\right). \end{array}$$

The input of the decoder consists of 3 parts [10], including the weighted sum of the output of the encoder *z*_*j*_, and the decision execution result of the previous step *s*_*j*−1_,*a*_*j*−1_. *z*_*j*_ is the context of the decoder in step *j*, which contains the attributes of the data to be sent.

Seq2seq neural network is an *n* -dimensional vector *d*. After nonlinear activation of this vector, an *n* -dimensional probability vector *π*_*θ*_ and a value function *v*, (*s*_*i*_), respectively, are obtained. The probability vector corresponds to *π*_*θ*_ the probability that the decision action takes a certain *a*, and the sum is 1. Then, the decision action of the *a*_*j*_=argmax_*a*_(*π*_*θ*_)*jth step* can be obtained through the greedy algorithm.

### 6.2. Parameter Update and Optimal Decision

To update the neural network parameters, this paper adopts the PPO2 [11] algorithm (Proximal Policy Optimization, proximal policy optimization). The remote transmission equipment may send 1 or more samples per day, and the daily objective function can be defined as

(15) $$\begin{array}{c} J={\text{ER}}_{\text{day}}. \end{array}$$

Based on the sequence representation of neural network, the objective function *J* for each sample (*θ*_*m*_) can be rewritten as

(16) $$\begin{array}{c} J\left(\theta_{m}\right)=E_{\theta }\left(R_{\text{day}}\left(\theta_{m}\right)\right). \end{array}$$

According to the law of large numbers,

(17) $$\begin{array}{c} J\left(\theta_{m}\right)\approx \sum_{m=1}^{M}\left(R\left(\theta_{m}\right)\right)=E\left(-W_{\text{day}}\right), \end{array}$$

where *M* is the number of samples, the data volume of each sample is divided into *n* data packet sequences and sent, *m* is the sample sequence number, that is, the data packet sequence. *R*(*θ*_*m*_) is the power consumption of each sample, namely,

(18) $$\begin{array}{c} R\left(\theta_{m}\right)=\sum_{i=1}^{n}\min\left(\omega_{i}*A_{i},{\text{clip}}_{1-\Delta }^{1+\Delta }\left(\omega_{i}\right)*A_{i}\right), \end{array}$$

Δ is the clipping constant, and the trend function [12] is

(19) $$\begin{array}{c} A_{i}=\sum_{j=0}^{n-i+1}{\left(\gamma *\omega_{i}\right)}^{j}*\left(r_{i+j}+\gamma *v_{\pi }\left(s_{i+j+1}\right)-v_{\pi }\left(s_{i+j}\right)\right). \end{array}$$

Among them, *γ* is the advantage function discount coefficient, *r* is the immediate return, and *v*_*π*_ is the state value function. *ω* are the adjustment coefficients for variance and bias,

(20) $$\begin{array}{c} \omega_{i}=\frac{\pi_{\theta_{m}}\left(a_{i}|s_{i}\right)}{\pi_{\theta_{m}^{0}}\left(a_{i}|s_{i}\right)}. \end{array}$$

According to the formula (10), *t*_*i*+*j*_ the immediate reward function that can be obtained is

(21) $$\begin{array}{c} r_{i+j}=\;E\left(-W_{i+j}\right). \end{array}$$

gradient update formula of the neural network parameters is

(22) $$\begin{array}{c} \theta_{i}^{\prime }=\theta_{i}+\alpha *\nabla_{\theta_{i}}\left(J\left(\theta_{m}\right)\right). \end{array}$$

Among them, *α* is a training coefficient, which is the learning rate, that is, the gradient descent factor. When the neural network parameters converge iteratively, the output of seq2seq is the optimal decision.

## 7. Experiment and Result Analysis

The list of simulation parameters used in this paper is as follows, including model parameters and algorithm parameters. In the simulation process, a sample is divided into 10, 12, 15, and 20 data packet sequences for training. In order to reduce the channel correlation, the minimum delay time segment of data packet transmission is taken as 5 s.

The water model and neural network, as well as the parameter settings of PPO2 are shown in Table 1.

**Figure 2:**
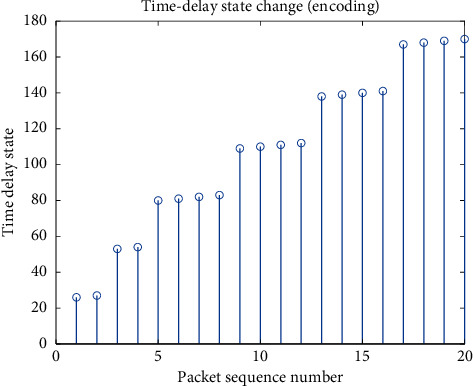
Delay change process of packet sequence.

**Figure 3:**
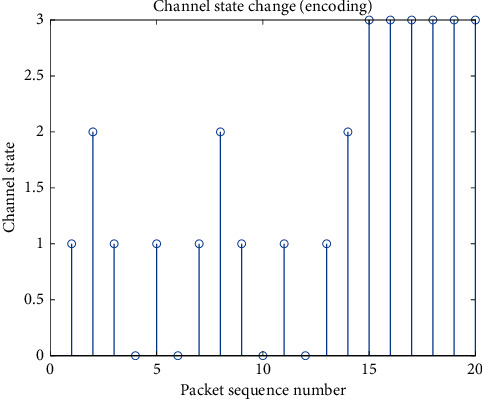
Channel change process of packet sequence.

After encoding the delay state and channel state, respectively, they are input into the neural network.  Figure 2 shows the change of the device delay state. When the decision is to send immediately, the delay is much smaller than the delay caused by suspending transmission.  Figure 3 shows the change of the channel state. The channel state, as the environment in which the smart device is located, is relatively stable when the signal-to-noise ratio is good, and is more likely to change randomly when the signal-to-noise ratio is poor.

**Figure 4:**
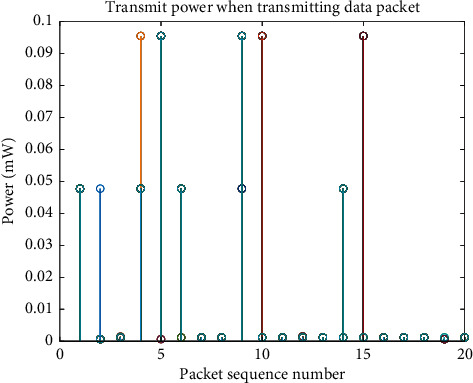
Transmit power of data packet sequence.

**Figure 5:**
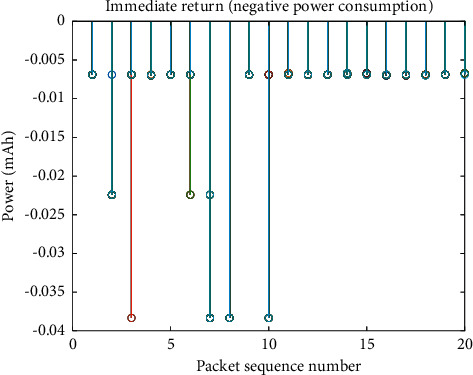
Immediate reward of packet sequence decision.

Each time the far-transmission device sends a data packet, it may either suspend the sending or send it immediately. The transmit power each time a data packet is sent is related to the state and environment it is in.  Figure 4 shows the power when different data packets are transmitted, and the different power levels reflect the equivalent model of repeated transmission. The power consumption of suspending the transmission of data packets is related to the idle current and the waiting time. The power consumption of sending a packet immediately is related to the transmit power/device voltage, and the transmit duration. The immediate return in Figure 5 is the negative value of power consumption, and the minimum power consumption is equivalent to the maximum return.

**Figure 6:**
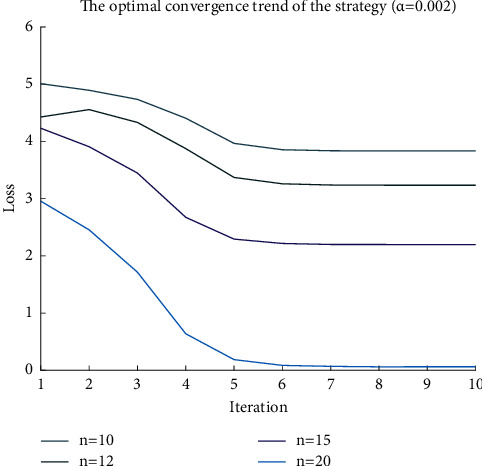
Convergence process of policy (learning rate 0.002).

**Figure 7:**
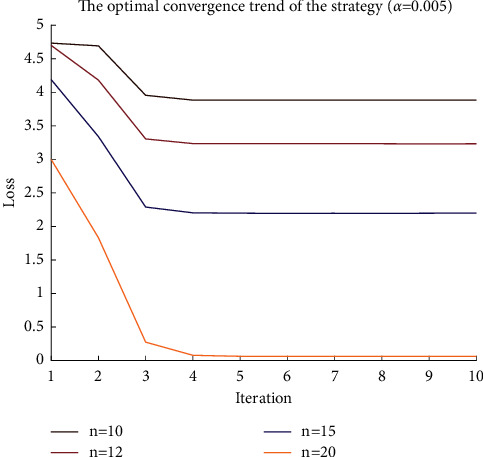
Convergence process of policy (learning rate 0.005).

After the water MDP model is established, it is solved by the PPO2 algorithm. For the sake of convenience, the reference objective function value is -6 in the experiment, and the difference between the training objective and the reference objective value is defined as the loss function.^.^ The parameters of the neural network converge as the number of iterations increases, and the strategy is also more optimized. When the loss function is stable, the parameters of the neural network converge to the optimal, and the corresponding strategy is the optimal expression of the neural network.  Figure 6 is the parameter iteration process when the learning rate is 0.002, and Figure 7 is the parameter iteration process when the learning rate is 0.0005. When the learning rate is increased, the convergence is faster, but the oscillation is also larger.

The agreement between the water remote transmission equipment and the platform stipulates that a single data should be less than 200 bytes, so when the amount of sample data to be sent is large, it will be split into more data packets. On the other hand, when the amount of data to be transmitted is constant, the more data packets are split, that is, the smaller the data packets, the lower the cost of retransmission, which is more suitable for the requirements of the narrowband Internet of Things. At this time, the loss function is smaller and easier to converge. Figures 6 and 7 are, respectively, the convergence of neural network parameters for 10, 12, 15, and 20 data packets.

**Figure 8:**
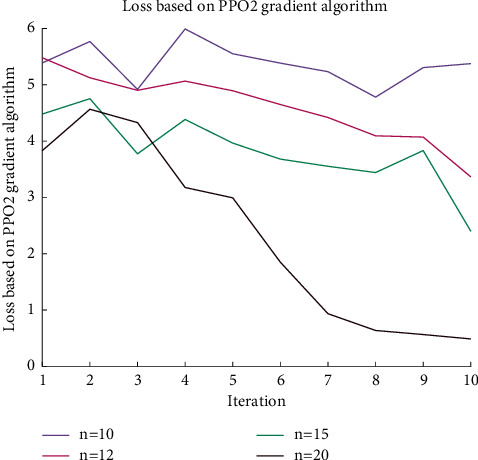
Convergence process with wider bias range (normal random distribution).

Figure 8 randomly distributes the full connection bias range of the neural network from 0 to 1. After adjusting to a normal random distribution, the convergence of the loss function will be more oscillating due to the increase in the dynamic range.

As mentioned above (Figure 5), if the perception model and algorithm in this paper are not used, the power consumption of one data packet is 0.04 mAh. If 20 data packets are sent every day and the life cycle of 10 years is calculated, the required battery capacity is 0.04 *∗* 20 *∗* 365 *∗* 10 =  2920 mAh. Generally, the medium battery capacity is 5000 mAh, which can support the device to send 1.71 samples per day, and each sample includes 20 data packets. After adopting the perception model and algorithm in this paper, the average power consumption of a data packet in Figure 5 is 0.018 mAh, which can support the device to send 3.7 samples per day. With the increase in the amount of data collected by equipment, the perception model and algorithm in this paper can support more installation point equipment with remote transmission units.

## 8. Conclusion

Starting from the actual problem of the layout and location mechanism of remote transmission equipment of intelligent water affairs, this paper establishes a system model based on MDP, namely, the Markov sequence decision model. The state, action, and reward function definitions of the model are given in this paper, and the simulation results of these definitions are given in the experimental part. The sequence decision is expressed based on the Seq2Seq neural network, and the PPO2 algorithm is used to solve the MDP sequence decision problem. The solution process and results are given in the experimental part. And when the model and method are applied to the actual water management problem, based on the life cycle of the remote equipment and the battery capacity value, the application scope of the equipment layout and location selection is solved.

## Figures and Tables

**Table 1:** Simulation parameters.

Parameter	Value description
Number of packets	*n* = {10, 12, 15, 20}
Suspend the smallest time segment	*δ* = 5 s
Packet transmission time	*T*_*f*_ = 1 s
Packet size	<200 bytes
SNR threshold	snr = [1.28 3.28 5.28 6.28]
Equipment voltage	*V* = 3
Device idle current	*I*_0_=250*μA*
Number of samples	*M* = 5
Expected life cycle	*d* = 10 *∗* 365 days
Learning rate	*α* = [0.002, 0.005]
Return discount factor	*γ* = 0.9 _
Advantage function discount factor	*ϕ* = 0.95
Clipping constant	Δ = 0.2
Number of neurons	unit *s* = 2 56;
Overfitting factor	0.5 _
Hidden unit	Layers = 256
Coding layer	Layer 1 = 2
Decoding layer	Layer 2 = 2

## Data Availability

The data used to support the findings of this study are available from the corresponding author upon request.
